# Cyclic Deformation Behavior of A Heat-Treated Die-Cast Al-Mg-Si-Based Aluminum Alloy

**DOI:** 10.3390/ma13184115

**Published:** 2020-09-16

**Authors:** Sohail Mohammed, Shubham Gupta, Dejiang Li, Xiaoqin Zeng, Daolun Chen

**Affiliations:** 1 Department of Mechanical and Industrial Engineering, Ryerson University, 350 Victoria Street, Toronto, Ontario M5B 2K3, Canada; sohail@ryerson.ca (S.M.); shubham.vaishya09@gmail.com (S.G.); 2 Department of Metallurgical and Materials Engineering, National Institute of Technology, Tiruchirappalli 620015, India; 3 State Key Laboratory of Metal Matrix Composites, School of Materials Science and Engineering, Shanghai Jiao Tong University, 800 Dongchuan Road, Shanghai 200240, China; xqzeng@sjtu.edu.cn

**Keywords:** AlMgSiMnFe alloy, heat treatment, low-cycle fatigue, cyclic hardening, serrated flow

## Abstract

The purpose of this investigation was to study the low-cycle fatigue (LCF) behavior of a newly developed high-pressure die-cast (HPDC) Al-5.5Mg-2.5Si-0.6Mn-0.2Fe (AlMgSiMnFe) alloy. The effect of heat-treatment in comparison with its as-cast counterpart was also identified. The layered (α-Al + Mg_2_Si) eutectic structure plus a small amount of Al_8_(Fe,Mn)_2_Si phase in the as-cast condition became an in-situ Mg_2_Si particulate-reinforced aluminum composite with spherical Mg_2_Si particles uniformly distributed in the α-Al matrix after heat treatment. Due to the spheroidization of intermetallic phases including both Mg_2_Si and Al_8_(Fe,Mn)_2_Si, the ductility and hardening capacity increased while the yield stress (YS) and ultimate tensile strength (UTS) decreased. Portevin–Le Chatelier effect (or serrated flow) was observed in both tensile stress–strain curves and initial hysteresis loops during cyclic deformation because of dynamic strain aging caused by strong dislocation–precipitate interactions. The alloy exhibited cyclic hardening in both as-cast and heat-treated conditions when the applied total strain amplitude was above 0.4%, below which cyclic stabilization was sustained. The heat-treated alloy displayed a larger plastic strain amplitude and a lower stress amplitude at a given total strain amplitude, demonstrating a superior fatigue resistance in the LCF regime. A simple equation based on the stress amplitude of the first and mid-life cycles (${\left(\Delta \sigma /2\right)}_{first}$, ${\left(\Delta \sigma /2\right)}_{mid}$) was proposed to characterize the degree of cyclic hardening/softening (*D*): $D=\pm \frac{{\left(\Delta \sigma /2\right)}_{mid}\;-\;{\left(\Delta \sigma /2\right)}_{first}}{{\left(\Delta \sigma /2\right)}_{first}},$ where the positive sign “+” represents cyclic hardening and the negative sign “−“ reflects cyclic softening.

## 1. Introduction

Aluminum alloys have been widely accepted as a key type of engineering materials, being particularly important for reducing weight, improving fuel efficiency, and diminishing environment-damaging, climate-changing and human death-inducing emissions (According to *Science News* entitled “Air pollution kills 7 million people a year” on March 25, 2014 at http://www.sciencemag.org/news/2014/03/air-pollution-kills-7-million-people-year: “Air pollution isn’t just harming Earth; it’s hurting us, too. Startling new numbers released by the World Health Organization today reveal that one in eight deaths are a result of exposure to air pollution. The data reveal a strong link between the tiny particles that we breathe into our lungs and the illnesses they can lead to, including stroke, heart attack, lung cancer, and chronic obstructive pulmonary disease.”; and recent *Science News* entitled “Air pollution is triggering diabetes in 3.2 million people each year” on July 9, 2018 at https://www.sciencenews.org/article/air-pollution-triggering-diabetes-in-millions-each-year: “Fine particulate matter, belched out by cars and factories and generated through chemical reactions in the atmosphere, hang around as haze and make air hard to breathe. Air pollution has been linked to chronic conditions such as heart disease and diabetes (SN: 9/30/17, p.18).” The findings reveal that “air pollution [is] responsible for about 14 percent of new cases of diabetes worldwide.” “The World Health Organization estimates that 422 million people now live with type 2 diabetes — up from 108 million in 1980.”) A significant portion of these emissions is caused by the transportation sector [1,2,3,4,5,6,7]. It has been reported that 10% of weight reduction in vehicle improves about 6–8% in the fuel efficiency [8,9]. Several high-strength and high-integrity Al-Si-Cu and Al-Mg-Si die-cast aluminum alloys have thus been developed for the automotive applications [10]. The increasing applications of lightweight Al-Si cast alloys lead to the development of novel or modified Al-Mg-Si alloys, since the inferior ductility and toughness of normal cast aluminum alloys failed to meet rigorous requirements of structural component subjected to cyclic stresses due to the existence of impurities and casting defects [11,12]. Therefore, die-cast aluminum alloys with better performance are required for high-integrity automotive components subjected to cyclic stresses. Die-cast Al-5.5Mg-2.5Si-0.6Mn-0.2Fe (AlMgSiMnFe) alloys studied in this work are expected to be competent to produce automotive structural components, thus providing more choices for automotive designers. It is thus essential to understand the fatigue behavior of such aluminum alloys to make its accessibility in the transportation sectors [13,14].

Although several studies involved the static and cyclic behavior of Al-Mg-Si alloys, there is not enough information reported on modified Al-Mg-Si alloys. Typically, Al-Mg-Si alloy contains primary α-Al, magnesium silicide (Mg_2_Si) and AlFeSi phases during solidification [15]. Recently, the authors presented the low-cycle fatigue (LCF) characteristics of cast AlMgSiMnFe alloy [16], but it is unknown how heat treatment would affect the microstructure and fatigue behavior. The non-equilibrium nature of solidification (i.e., high cooling rates, pouring velocities and nucleation) leads to micro-segregation and formation of a wide range of intermetallic phases [15,17]. Thus, homogenization via heat treatment of such cast alloys becomes necessary to modify the morphology and type of intermetallics. In particular, the brittle and interconnected eutectic Mg_2_Si phase is commonly observed in the hypoeutectic [18], eutectic [19] and hypereutectic [20] compositions and provides crack initiation sites and an easy path for propagation [18]. Hence, the eutectic phase is being altered by chemical modification and heat treatment.

In the present study, the alloy chemical composition is optimized by taking into consideration: (1) the weight fraction of impurities is reduced to a low level. (2) The weight ratio of Mg/Si is maintained at ~2.2 to prevent the hot tearing tendency and obtain 40–50% of the eutectic structure. (3) Fe content is reduced to as low as 0.2% to avoid its detrimental effect of crack initiation sites. (4) Si (2.5%) and Mn (0.6%) are added to facilitate die-casting process by means of improving the formability and decreasing sticking between mold and casting. The microstructure of the heat-treated alloy is characterized via scanning electron microscope (SEM) and electron backscatter diffraction (EBSD). LCF tests are performed on the heat-treated alloy and compared with the as-cast counterpart. The strain–fatigue life relationship of the alloy is also evaluated. Dynamic strain aging (DSA) observed during the tensile tests and in the initial stress–strain hysteresis loops during cyclic deformation is assessed and linked to the microstructure.

## 2. Experimental Procedure

### 2.1. Material

The material used in this study was Al-5.5Mg-2.5Si-0.6Mn-0.2Fe (abbreviated as AlMgSiMnFe) produced by high pressure die-casting (HPDC). Traces of about 0.1% La/Ce were added in the alloy to avoid melt oxidation. The Mg content of more than 3.5% was chosen to minimize hot tearing tendency, since the hot tearing of alloys tended to become more serious with a lower amount of Mg. Thus, such a cast alloy is prominent in castibility, fluidity, hot tearing resistance along with good strength and ductility. The alloy selected in this study was further heat-treated in T6 condition as specified in [21]. That is, it was solution treated at 500 °C for 2 h, then water cooled, followed by aging at 180 °C for 10 h, then air cooling to room temperature. Prior to the heat treatment, calibration of furnaces was carried out to ensure the accuracy of temperatures.

### 2.2. Microstructure Characterization

Optical microscope (OM) images were taken for the alloy in both as-cast and heat-treated conditions. The SEM images were obtained using SEM (JSM-6380LV, JEOL, Tokyo, Japan) equipped with an energy dispersive X-ray spectroscopy (EDS). The samples for OM and SEM observations were etched for 30 s using Keller’s reagent containing 2 mL HF, 3 mL HCl, 5 mL HNO_3_ and 190 mL H_2_O followed by ultrasonic cleaning. Prior to this, both as-cast and heat-treated samples were mechanically polished using SiC papers (#400, #600, #1200, #2000 and #4000), followed by polishing using diamond paste (1 and 0.5 μm) and then 0.05 μm colloidal silica. The samples for EBSD analysis were further electro-polished after mechanical polishing, using an electrolyte containing 10 mL nitric acid and 40 mL ethanol at 15 V for 15 s at room temperature. X-Ray Diffraction (XRD) analysis was performed from 20° to 90° (diffraction angle) at a scanning rate of 0.05° s^−1^ using X-ray diffractometer (PANalytical, Eindhoven, Netherlands) equipped with Cu K_α_ radiation at 45 kV and 40 mA.

### 2.3. Mechanical Testing

**Figure 1:**
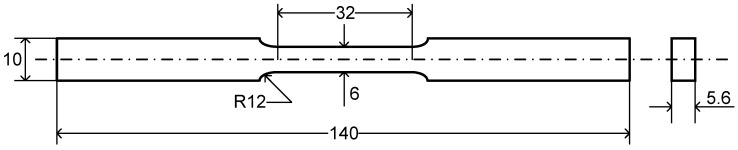
Geometry and dimensions of tensile and fatigue test specimens (all dimensions are in mm).

Hardness tests were carried out on the metallographic as-cast and heat-treated samples using a computerized microhardness tester (Mitutoyo, Tokyo, Japan) at a load of 200 g and a dwell time of 15 s. Five indentations were taken and the relevant average was presented. To ensure the accuracy of hardness values measured, a standard hardness block was used to calibrate the measurements prior to indenting the real samples. Tensile tests were performed on both as-cast and heat-treated specimens according to ASTM E8/E8M standards [22] at a strain rate of 1 × 10^−2^ s^−1^ at room temperature using a computerized universal United testing system. The geometry and dimensions of tensile and fatigue test specimens are shown in Figure 1. All specimens were mechanically ground along the loading direction using grit #600 SiC sandpapers to obtain consistent surfaces and avoid potential effects of stress raisers. The yield stress (YS), ultimate tensile strength (UTS), percent elongation (%El) and strain-hardening exponent (*n*) were evaluated from the tensile results by testing at least two samples in each case.

### 2.4. LCF Testing

LCF tests were performed under strain-controlled mode using Instron 8801 servo-hydraulic system (Instron, Norwood, MA, USA) at different strain amplitudes (0.1%, 0.2%, 0.4%, 0.6% and 0.8%) at a strain ratio of *R* = −1 and a strain rate of 1 × 10^−2^ s^−1^. To keep the constant strain rate, the frequency for the tests was varied from 0.3125 to 2.5 Hz for the total strain amplitudes of 0.8% to 0.1%. The fatigue tests at the strain amplitudes of 0.1% and 0.2% were initially performed under a strain-controlled mode and then transferred to a stress-controlled mode after 10,000 cycles were reached without failure, using a sinusoidal waveform at a frequency of 50 Hz. An extensometer with a gage length of 25 mm was mounted on the sample to control and monitor strains. Crack initiation sites and fracture mechanisms were then analyzed on the samples fatigued at a total strain amplitude of 0.2% and 0.6% through fractography.

## 3. Results and Discussion

### 3.1. Microstructure

**Figure 2:**
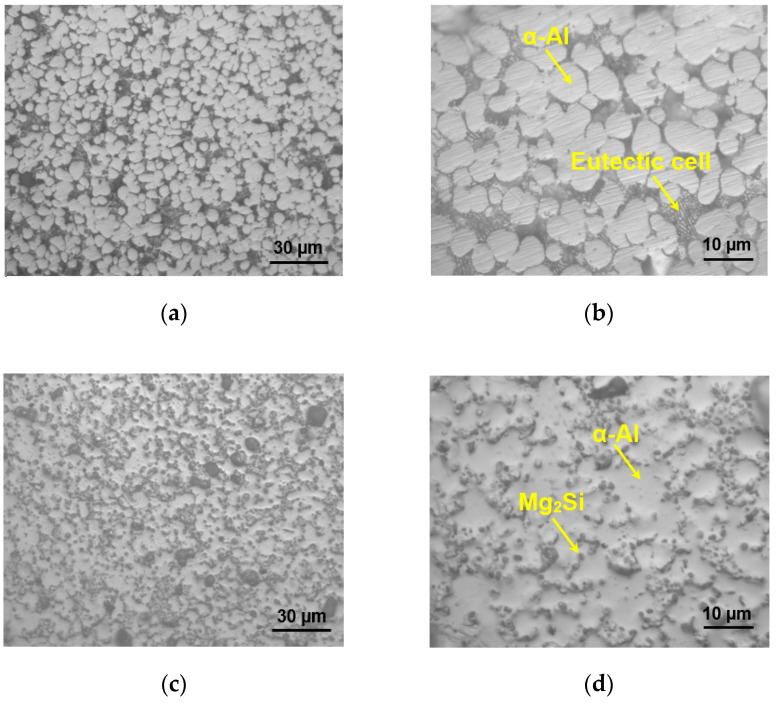
Typical optical microscope (OM) images showing the microstructures of as-cast Al alloy at (**a**) lower and (**b**) higher magnifications and heat-treated Al alloy at (**c**) lower and (**d**) higher magnifications.

The microstructure of the as-cast AlMgSiMnFe alloy consists of primary α-Al and eutectic structure (α-Al + Mg_2_Si) as shown in Figure 2a,b. This is related to the strong chemical affinity between the atoms of Mg and Si [23], along with the approximate 2:1 atom ratio of Mg to Si specifically designed in the present alloy. The inter-precipitate distance and density are strongly dependant on the ratio of Mg/Si, while the size of the precipitates is unaffected by the ratio [24]. After heat-treatment, the elongated eutectic Mg_2_Si layers or rods in the cast state become basically spheroidized, with many spherical Mg_2_Si particles embedded in α-Al phase mainly due to the solution heat-treatment at 500 °C as seen in Figure 2c,d. Similar spheroidization or short fiber-like and spherical morphologies of Mg_2_Si particles were also observed in an Al-10%Mg_2_Si alloy [25] and in an Al-20Mg_2_Si-4.5Cu alloy [26] after T6 heat treatment.

**Figure 3:**
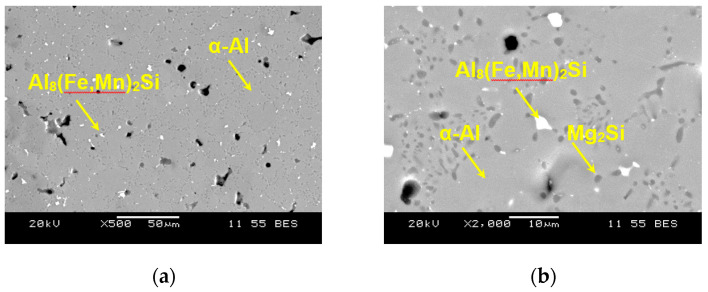
Typical SEM images showing the microstructure of heat-treated AlMgSiMnFe alloy at (**a**) lower (500×) and (**b**) higher (2000×) magnifications.

**Figure 4:**
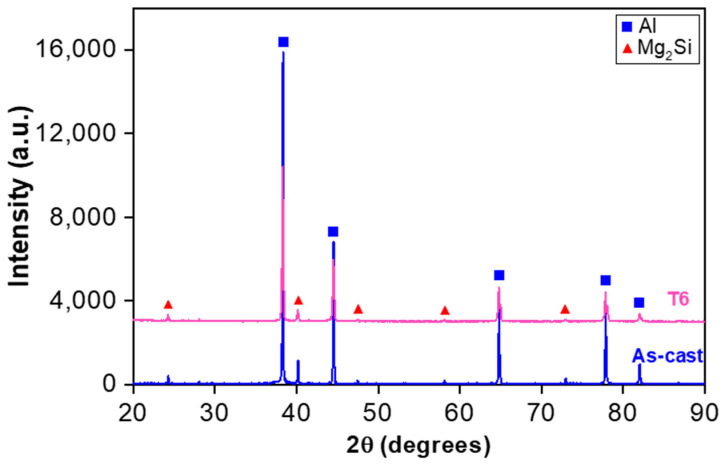
XRD patterns of the heat-treated and as-cast alloys.

As seen from the SEM backscattered electron (BSE) images (Figure 3), the microstructure of heat-treated sample consists particularly of spheroidized Mg_2_Si along with some white Al_8_(Fe,Mn)_2_Si particles, which are mainly distributed along the grain boundaries and triple joint junction. Generally, these Fe-rich intermetallics, e.g., Al_8_Fe_2_Si with a Chinese script morphology [27,28], are easily formed at the grain boundaries and triple point junction, and may act as stress concentration point which promotes crack initiation and deteriorates ductility and fatigue characteristics. Hence, the amount of Fe should be kept small. However, in the present alloy the long needle-shaped *β*-AlFeSi phases, which were observed to reduce the ductility, have changed their morphology to small spherical particles due to a small (0.2%) amount of Fe and heat treatment. Thus, the ductility is significantly improved, as seen in Table 1, although the strength in the heat-treated condition decreases in comparison with the as-cast condition. This is mainly attributed to the spheroidization of both types of intermetallic phases (Mg_2_Si and Al_8_(Fe,Mn)_2_Si) in the heat-treated condition, being similar to the spheroidization phenomenon of cementite (or iron carbide Fe_3_C) in pearlitic steels to form the softer and more ductile spheroidite. The EDS analysis of the as-cast alloy can be found elsewhere [16]. Magnesium content in general is primarily responsible for high strength-to-weight ratio, as Mg combines with other elements to form second-phase particles, e.g., Mg_2_Si participles [29,30]. Figure 4 illustrates the presence of α-Al and Mg_2_Si phases via XRD analysis of the alloy in both as-cast and heat-treated conditions. It is clear that the Mg_2_Si phase in the alloy is still retained after heat treatment, as the melting temperature of Mg_2_Si is about 1085 °C [31,32].

### 3.2. EBSD Analysis

**Figure 5:**
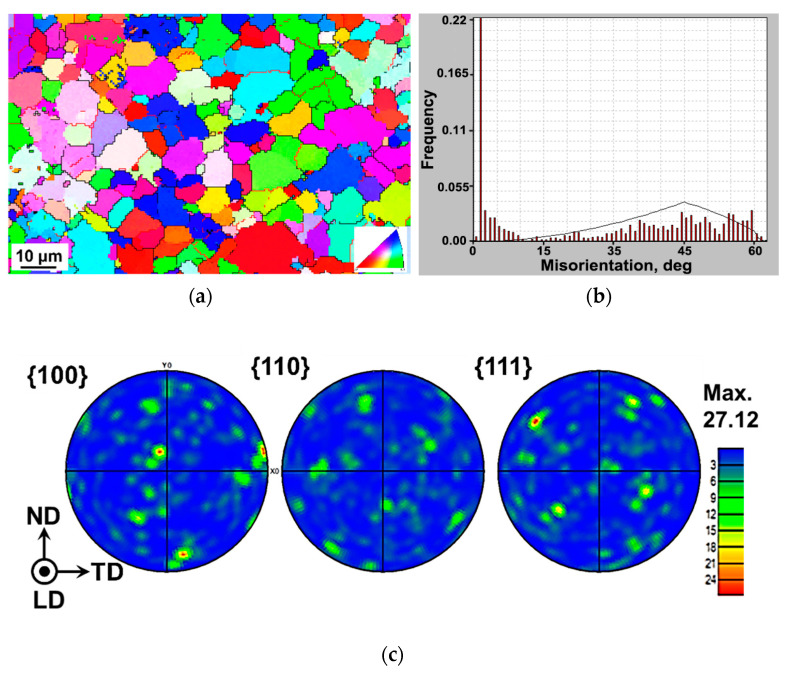
(**a**) Electron backscatter diffraction (EBSD) orientation map, (**b**) grain boundary misorientation angles, (**c**) {100}, {110} and {111} pole figures of the alloy in the heat-treated condition.

Figure 5 shows the EBSD orientation maps of the alloy in the heat-treated condition. The sample was observed along the longitudinal direction which is also the melt flow direction of the casting. The maps show that there was no significant preferential orientation (or texture) in the specimen. The grains were mostly equiaxed and randomly oriented. The angles between 2° and 15° are defined as low-angle grain boundaries (LAGBs) and marked in red lines, whereas the angles above 15° are classified as high-angle grain boundaries (HAGBs) and marked in black lines. It is evident that the alloy contains more HAGBs. There were barely any non-indexed regions (black spots in Figure 5a) usually caused by the presence of pores in cast alloys which indicates that the amount of porosity was minimal in the alloy. Figure 5b shows the histogram of misorientation angles versus relative frequency. The misorientation angle of a large number of grains is seen to be lower than 5°. The average grain size of the alloy in the heat-treated condition becomes larger in comparison with that in the as-cast condition [16]. Three pole figures (i.e., {100}, {110} and {111}) are calculated as shown in Figure 5c. A fairly random texture was observed in the material after heat treatment, being similar to that in the as-cast state [16].

### 3.3. Tensile Properties

**Figure 6:**
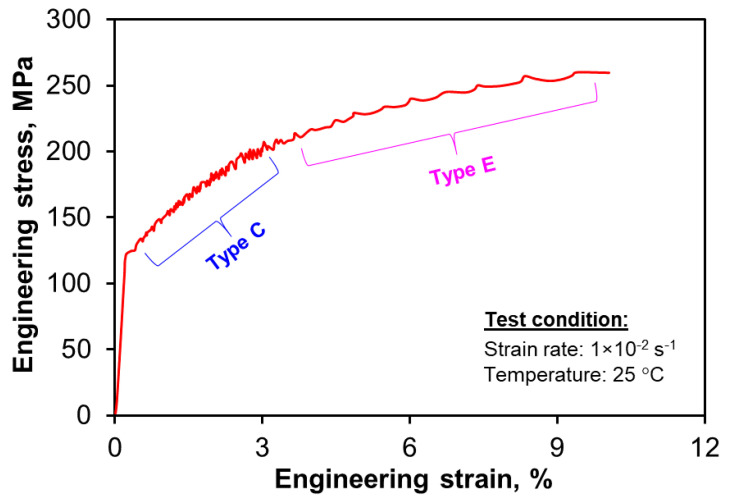
A typical tensile stress–strain curve of the alloy in the heat-treated condition.

The tensile tests of the present alloy in the heat-treated state were carried out at a strain rate of 1 × 10^−2^ s^−1^ at room temperature. It was observed that the yield strength decreased from 185 MPa in the as-cast condition to 122 MPa in the heat-treated condition, while the ductility increased significantly from 6.3% to 15.7%, as shown in Table 1 and Figure 6. A similar decrease in strength in an Al-5Mg alloy with varying Si contents was reported by Johannesson and Caceres [33], who reported a decrease in the yield strength from 106.5 to 88 MPa and from 80 to 60 MPa from the as-cast state to T6 state in Al-5Mg-1Si and Al-5Mg-3Si alloys, respectively. Besides, the present HPDC alloy exhibited a much higher strength and ductility than the similar sand cast Al-5Mg-3Si alloy in both as-cast and heat-treated conditions. A possible reason for the effect of T4 or T6 heat treatment was noted to be related to the fragmentation of intermetallic particles imposed on the matrix [33]. In this study, the spheroidization of intermetallic phases including both Mg_2_Si and Al_8_(Fe,Mn)_2_Si in the heat-treated condition, as discussed above and shown in Figure 2d and Figure 3b would be the main reason; this is in sharp contrast to the layered eutectic structure in the as-cast alloy [16]. With the vanishing of a layered eutectic structure and the spheroidization of Fe-rich intermetallics, the resistance of spherical particles to the movement of dislocations becomes lower. Thus there was a decrease in the strength of heat-treated alloy. The hardening capacity, $H_{c}$, defined by Afrin et al. [34] can be expressed as follows:

(1) $$H_{c}=\;\frac{\sigma_{UTS}-\;\sigma_{y}}{\sigma_{y}}$$

where $\sigma_{UTS}$ and $\sigma_{y}$ are UTS and YS of the material, respectively. The present heat-treated alloy shows a higher hardening capacity of 1.13 (Table 1), compared to the as-cast counterpart, being 0.72 obtained at same strain rate [16]. The following Holloman equation is used to evaluate the strain hardening exponent (*n*),

(2) $$\sigma =K\varepsilon^{n}$$

where σ, *K* and ε are true stress, strength coefficient and true strain, respectively. The obtained *n* value is slightly higher in the heat-treated condition than in the as-cast condition, as shown in Table 1. The microhardness value of the alloy in the heat-treated condition (71.4 ± 2.7 HV) was obtained to be lower than that of the as-cast alloy (92.7 ± 1.6 HV). The softening of the alloy in the heat-treated condition is again related to the spheroidization of eutectic structure and fragmentation of intermetallic particles which lead to improved ductility. The solution temperature and time has a strong influence on hardness value [35]. Furthermore, serrated flow or Portevin–Le Chatelier effect of type C was observed in the flow curve after yielding, followed by type E beyond a strain of ~4% as a result of DSA [36,37]. The serrations caused by DSA fundamentally resulted from the interaction of diffusing solute atoms (mainly Mg in this case) and mobile dislocations, thus raising the activation energy for continued slip during plastic deformation [36,37,38]. A stress drop occurs at a point when the material reaches a level to cause the motion of pinned dislocations, followed by the intense dislocation activity until the dislocations are re-pinned [39].

### 3.4. Fatigue Behaviour

**Figure 7:**
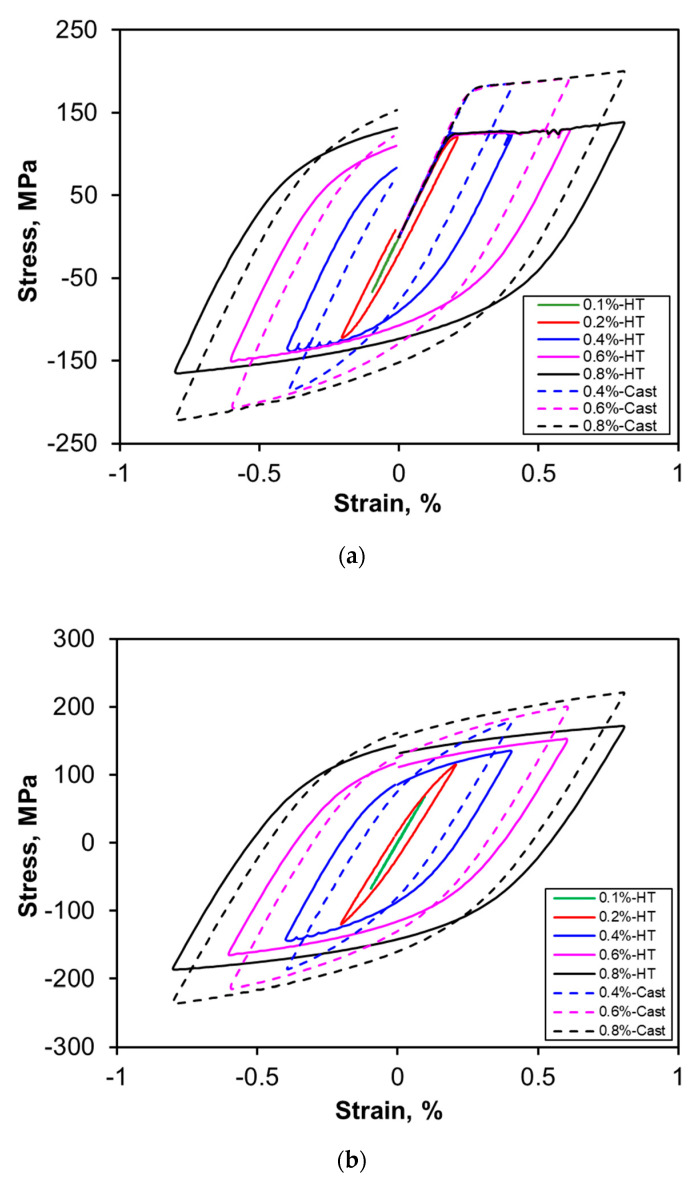

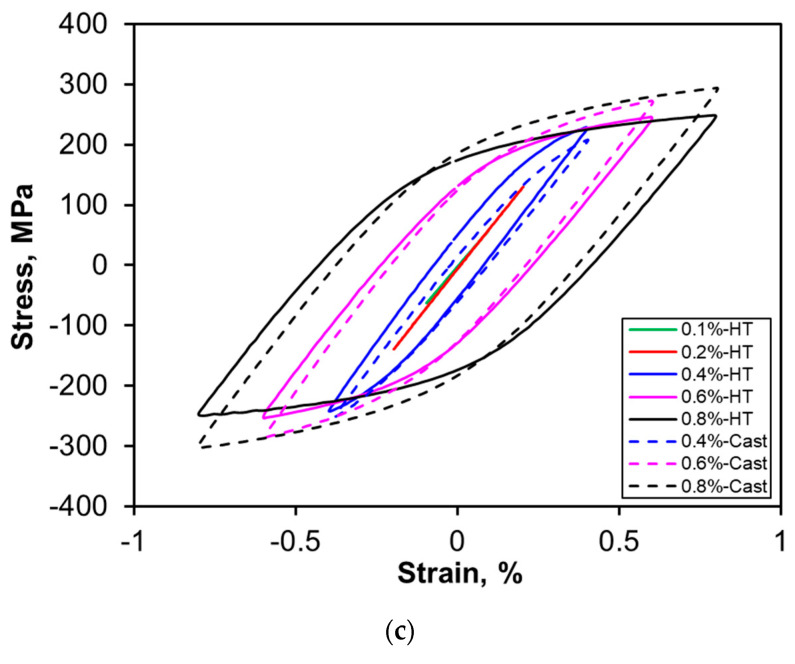
Typical hysteresis loops of the alloy in the as-cast and heat-treated states at various total strain amplitudes for the (**a**) first, (**b**) second, and (**c**) mid-life cycles.

Figure 7a–c shows the hysteresis loops for the first, second and mid-life cycles of the heat-treated specimens at strain amplitude 0.4%, 0.6% and 0.8%, respectively, in comparison with those of as-cast specimens [16]. Unlike the situation in the extruded magnesium alloys [40,41,42], the hysteresis loops here are basically symmetrical in nature, as also reported in the literature for several other aluminum alloys [13,43,44]. Apparently, the peak stress in tension is very similar to the minimum stress in the hysteresis loops, which indicates that there is no tension-compression asymmetry. It is seen that the initial opening quarters of the hysteresis loops are overlapped nicely at different strain amplitudes, which can be used to calculate the yield strength (YS) of the material [45]. Using the first-quarter cycle of hysteresis loop at a strain amplitude of more than 0.4%, the YS evaluated is 127 MPa, which is very close to the monotonic YS (122 MPa) at the same strain rate (1 × 10^−2^ s^−1^). The width of stress–strain hysteresis loops increases with increasing strain amplitude from 0.1% to 0.8% and is also larger than that of the as-cast alloy in general at all strain amplitudes, as seen from Figure 7. The increase in the width of stress–strain hysteresis loops is directly related to the superior ductility in the heat-treated alloy (Table 1) arising mainly from the occurrence of spheroidization of Mg_2_Si-containing eutectic structure (Figure 2d and Figure 3b).

Furthermore, serrated flow was also observed to appear in the plastic regime in the compressive phase at the strain amplitudes of 0.4% and beyond. A couple of serrations at irregular intervals were also observed in the initial tensile phase at total strain amplitudes from 0.4% to 0.8%, as shown in Figure 7a. This is in agreement with the observation of serrated flow, resulting from DSA, during the monotonic tensile test of the heat-treated alloy subjected to much higher level of straining. During LCF, the serrations were basically observed in the initial (first and second) cycles, then gradually disappeared at mid-life where stabilization was attained as can be seen from Figure 7c. The disappearance of serrations after prolonged cycles has been reported to be attributed to the diffusion of solute atoms through dislocations to the precipitate sinks, which in turn leads to the disappearance of serrations [46,47]. The serrations on the cyclic hysteresis loops were found to be type C in nature regardless of strain amplitude, corresponding well to the monotonic tensile test results (Figure 6). The magnitude of serration amplitude was found to be higher at lower strain amplitude (0.4%) and decrease with increasing strain amplitude. A similar serrated flow phenomenon present in the hysteresis loops was also reported during strain-controlled LCF of Al-Zn-Mg alloy in a natural aging condition [39] and in other materials, e.g., annealed Zircaloy-2 [46], forged alloy 617M in the solution annealed condition [47], Inconel 718 superalloy [48], duplex Ti-6Al-4V and SP700 titanium alloys [49], and extruded AM50 magnesium alloy [50]. It should be noted that the serrated flow behavior is dependent on the strain rate and temperature [51,52,53]. Recently, Mohammed and Chen [52] observed that the serrations during uniaxial tensile testing disappeared after all the three (displacement, load, and strain) channels in the servo-hydraulic fatigue machine were auto-tuned due to the shielding effect for control stability. However, in the present study the serrations are still observed even after auto-tuning of the strain channel. This is likely due to a very strong effect of dislocation–precipitate interactions, i.e., the role of DSA during LCF [36], and the presence of numerous dissolved Mg atoms in the present alloy after heat treatment which could be further confirmed by Masing behavior study.

**Figure 8:**
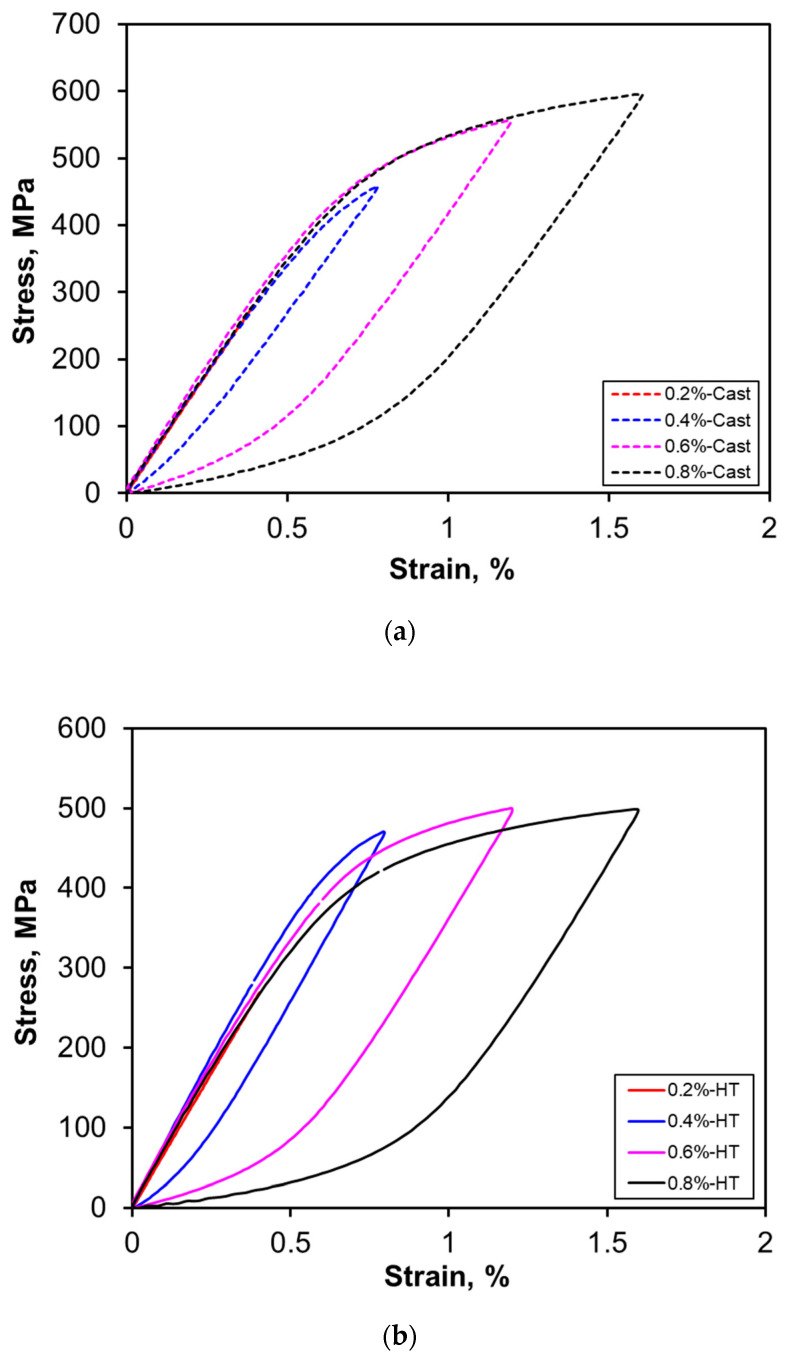
Typical hysteresis loops at various total strain amplitudes of AlMgSiMnFe alloy, showing (**a**) Masing behavior in the as-cast alloy and (**b**) non-Masing behavior in the HT alloy.

The hysteresis loops corresponding to the mid-life (*N_f_*/2) cycle at different strain amplitudes are plotted by translating all the loops with the minimum peak positioned at the origin [54], as shown in Figure 8a,b for the alloy in both as-cast and heat-treated conditions, respectively. It is evident that the as-cast alloy exhibits Masing-like behavior, which is absent after heat treatment. Christ and Mughrabi [55] reported that Masing behavior can be observed if dislocation–dislocation interactions play a more important role than dislocation–precipitate interactions. This corresponds well to the present heat-treated alloy, which possesses more precipitates as compared to the as-cast condition, leading to more pronounced dislocation–precipitate interactions. This is also in agreement with the presence of serrated flow behavior caused by the DSA during the initial cycles in the heat-treated alloy (Figure 7a,b).

**Figure 9:**
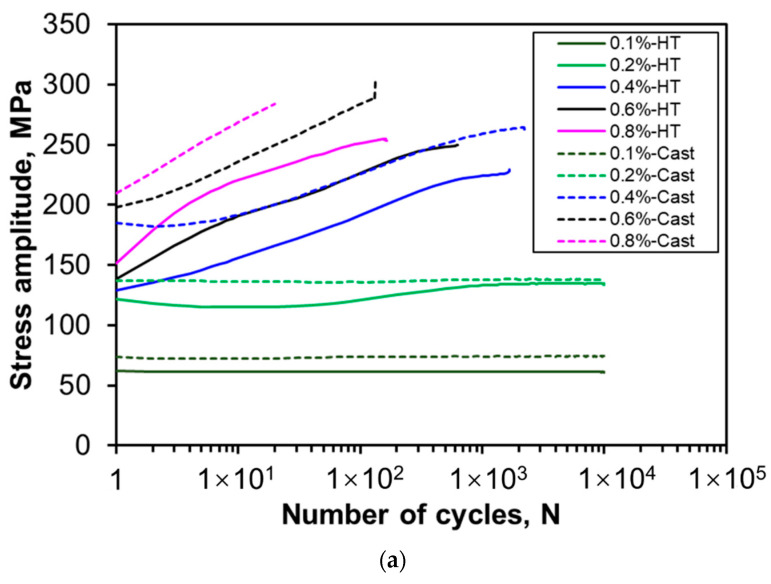

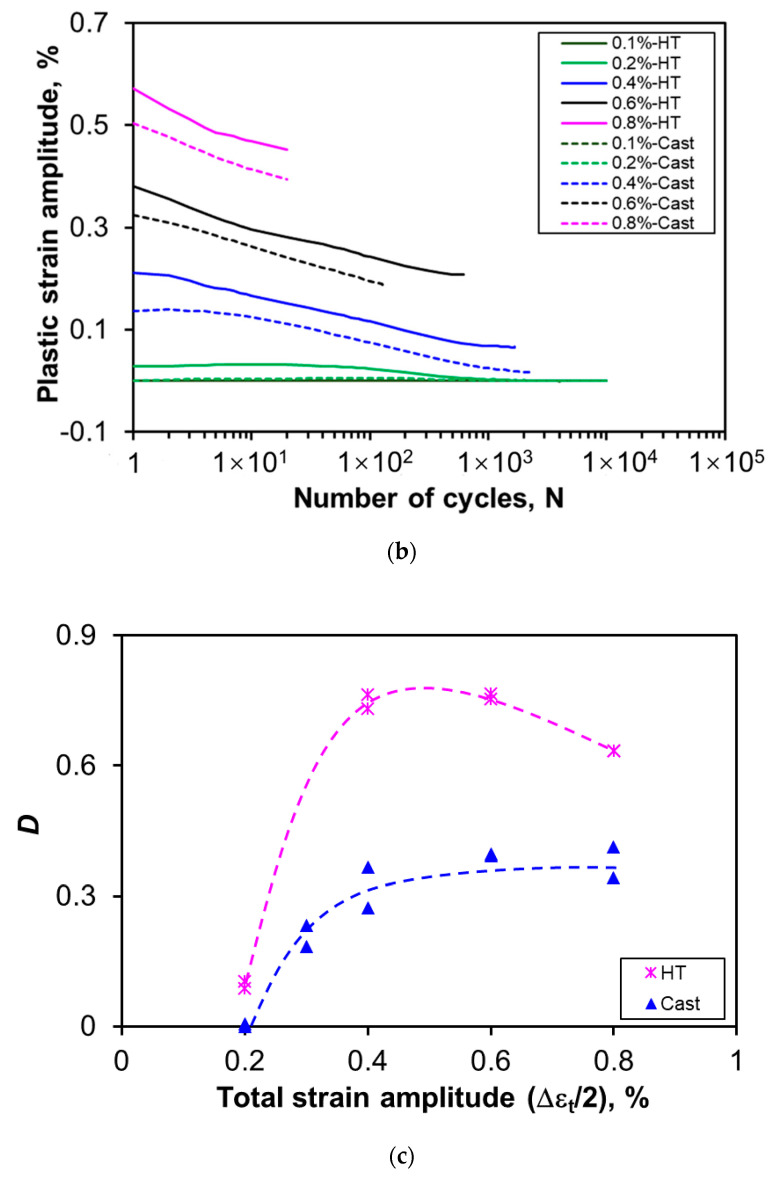
(**a**) Cyclic stress amplitude and (**b**) plastic strain amplitude as a function of number of cycles, and (**c**) newly-defined degree of cyclic hardening (*D*) versus the total strain amplitude in both as-cast and heat-treated alloys tested at a strain ratio of *R_ε_* = −1.

Figure 9a shows the plot between the stress amplitude and the number of cycles at various strain amplitudes ranging from 0.1% to 0.8% on a semi-log scale. It is seen that a stable/constant stress amplitude remains at total strain amplitudes of 0.1% and 0.2%, while cycling hardening occurs at higher strain amplitudes (0.4–0.8%) in both heat-treated and as-cast conditions. The plastic strain amplitude is a widely accepted physical quantity to relate the internal microstructure of the alloy with the damage process related to fatigue resistance and fatigue life [56]. The change in the plastic strain amplitude during cyclic deformation is shown in Figure 9b, which demonstrates cyclic hardening behavior similar to the stress amplitude (Figure 9a) but in a reverse way. Similar cyclic hardening behavior has been reported in cast aluminum alloys by other authors [13,57,58]. It is also seen from Figure 9a,b that at a given strain amplitude, the stress amplitude is higher in the as-cast condition than in the heat-treated condition, while the plastic strain amplitude is just reversed, being in agreement with the tensile test results tabulated in Table 1. As mentioned earlier, the as-cast specimen consists of primary α-Al along with layered α-Al and Mg_2_Si eutectic microstructure, while the heat-treated specimen contains the spheroidized particles embedded in the α-Al phase (Figure 2d and Figure 3b). The hardening or softening during cyclic loading is dependant on the ratio of $\sigma_{UTS}$ and $\sigma_{y}$. If the ratio of $\sigma_{UTS}/\sigma_{y}$ is greater than 1.4, cyclic hardening dominates, while cyclic softening is expected to occur if the ratio is smaller than 1.2 [59]. In the present study, the degree of cyclic hardening or softening (*D*) based on the change of stress amplitude (Figure 9a) could be defined as,

(3) $$D=\;\pm \frac{{\left(\Delta \sigma /2\right)}_{mid}\;-\;{\left(\Delta \sigma /2\right)}_{first}}{{\left(\Delta \sigma /2\right)}_{first}}$$

where the positive sign “+” stands for cyclic hardening and the negative sign “-“ reflects cyclic softening, ${\left(\Delta \sigma /2\right)}_{first}$ and ${\left(\Delta \sigma /2\right)}_{mid}$ are the stress amplitude of the first cycle and mid-life ($N_{f}/2$) cycle, respectively. The values of *D* as a function of total strain amplitude ($\Delta \varepsilon_{t}/2$) are plotted in Figure 9c. Two stages could be seen: initially *D* increases up to a total strain amplitude of 0.4% followed by a plateau in the as-cast alloy, but by a slight decrease in the heat-treated alloy. However, the *D* value of the heat-treated alloy is much higher relative to the as-cast counterpart at all strain amplitudes applied. This indicates that the heat-treated alloy has a much higher cyclic hardening capacity, being in agreement with the monotonic hardening capacity (Table 1) arising from the initially softer and more uniform state. It is worth noting that the use of mid-life fatigue data in Equation (3) is based on the consideration from ASTM E606, where the mid-life hysteresis loop is recommended to represent a conditional “cyclic saturation” situation if no real cyclic saturation or stabilization occurs for some materials.

**Figure 10:**
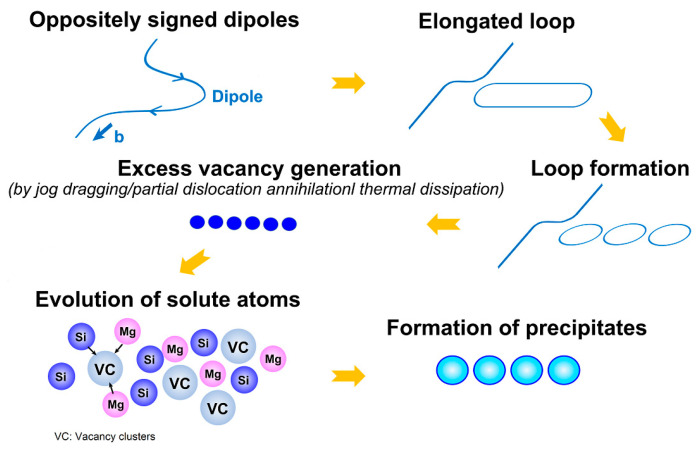
Schematic representation of formation of precipitates during cyclic deformation.

The cyclic hardening of Al-Mg-Si alloy in a relatively soft under-aging state is due to the presence of Guinier–Preston (GP) zones and Mg_2_Si particles (Figure 2d and Figure 3b). Several authors [39,60,61] have suggested different mechanisms to explain the cyclic hardening in under-aged Al alloys during LCF, including the formation of GP zones [60], vacancy driven over-aging of GP zones [61] and dynamic strain aging [39]. Hence, it could be presumed that the cyclic hardening of the present alloy is a result of either cyclic strain-induced precipitation or the formation of particular dislocation structure caused by DSA as revealed by the serrated flow (Figure 6 and Figure 7). Dynamic precipitation is reported to occur in some under-aged Al alloys during cyclic loading due to the formation of vacancies. It is also known that a large number of vacancies are generated by plastic deformation, which leads to the formation of GP zone. The vacancies are generated due to the formation of dislocation dipole loops which follows sequential events of jog dragging, thermal dissipation of vacancies and partial dislocation annihilation [60]. The formation of prismatic loops from elongated loops is caused by negative climb. During monotonic loading at room temperature, it is not possible for the vacancies to sink but cyclic straining increases the concentration of vacancies [62]. The process of generation and emission of vacancies is schematically illustrated in Figure 10. The deformation behavior is further characterized by fatigue life analysis in the next section.

### 3.5. Fatigue Life Analysis

**Figure 11:**
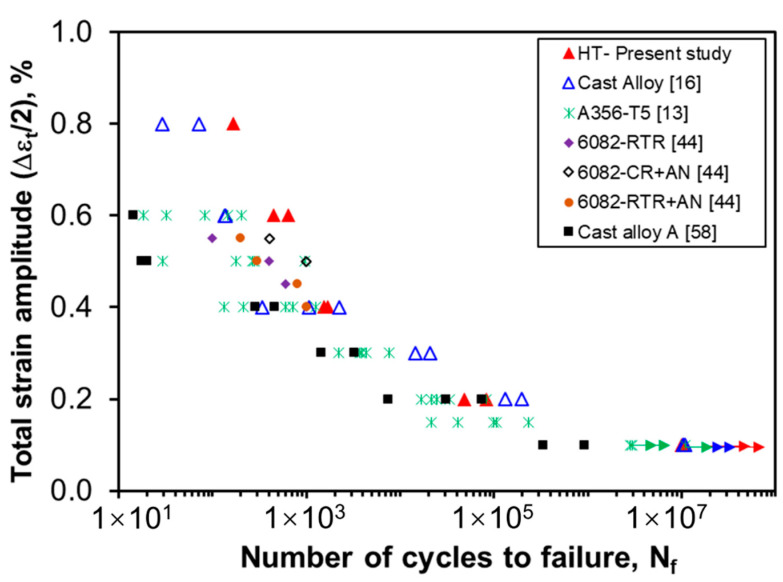
Fatigue life of heat-treated Al alloy in comparison with that of other alloys reported in the literature.

Figure 11 presents a summary of LCF fatigue life of the heat-treated alloy under strain-controlled mode. It is evident that the heat-treated alloy shows a longer fatigue life at higher strain amplitudes as compared with the as-cast alloy. At the lower strain amplitude of 0.1%, both heat-treated and as-cast samples did not fail even after 10^7^ cycles, which are indicated with horizontal arrows. In general, the LCF life is related to both strength and ductility of the material. The heat-treated alloy exhibits improved ductility along with a lower strength compared with the as-cast alloy, which leads to better fatigue resistance at higher strain amplitudes. The fatigue life of the alloy is also compared to its as-cast condition and other commercial aluminum alloys [13,16,44,58]. The present alloy basically exhibits a higher fatigue resistance compared with several other aluminum alloys. One possible reason behind this is due to the presence of Mg_2_Si particles (Figure 2d and Figure 3b), rather than Si particles in the normal Al-Si cast alloys, since Si particles are broken [63,64] or deformed relatively easily, e.g., by twinning [65]. The small amount (0.2%) of Fe has also been reported to increase the fatigue life of the present alloy by forming a small amount of Fe-containing intermetallics [43]. Furthermore, Hu et al. [66] reported that the fatigue life increases with increasing content of magnesium as well, which is ~5.5 wt.% in the present alloy.

The fatigue life of a material could be described by the Basquin’s equation and Coffin–Manson relationship for the high-cycle and low-cycle fatigue lives, respectively. The total strain amplitude, $\Delta \varepsilon_{t}/2$, by combining both Basquin’s and Coffin–Manson equations, is given as [67]:

(4) $$\frac{\Delta \varepsilon_{t}}{2}=\;\frac{\Delta \varepsilon_{e}}{2}+\;\frac{\Delta \varepsilon_{p}}{2}=\;\frac{\sigma_{f}^{\prime }{\left(2N_{f}\right)}^{b}}{E}+\;\varepsilon_{f}^{\prime }{\left(2N_{f}\right)}^{c}$$

where ∆ε/2 is the strain amplitude with suffix *e* and *p* representing elastic and plastic components, respectively; *E* is the Young’s modulus, 2*N*_f_ is the number of reversals to failure, *σ*‘_f_ is fatigue strength coefficient, *b* is the fatigue strength exponent which is also known as Basquin exponent, *ε‘_f_* and *c* are fatigue ductility coefficient and exponent, respectively. Table 2 shows the calculated values of the fatigue strength exponent and fatigue ductility exponent of the alloy in the heat-treated condition compared with the as-cast alloy. The values of *b* and *c* are also assessed using Morrow [68] and Tomkins [69] equations as given below:

Morrow:

(5) $$b\;\approx \;\frac{-n^{\prime }}{1+5n^{\prime }}\;;\;c\;\approx \;\frac{-1}{1+5n^{\prime }}$$

Tomkins:

(6) $$b\;\approx \;\frac{-n^{\prime }}{1+2n^{\prime }}\;;\;c\;\approx \;\frac{-1}{1+2n^{\prime }}$$

where $n^{\prime }$ is the cyclic hardening exponent. The estimated *b* and *c* values are also tabulated in Table 2. The cyclic stress–strain curve (CSSC) is also a key characteristic in the fatigue properties like the monotonic tensile stress–strain curve, and it could be expressed as follows [40]:

(7) $$\frac{\Delta \sigma }{2}=\;K^{\prime }{\left(\frac{\Delta \varepsilon_{p}}{2}\right)}^{n^{\prime }}$$

where Δσ/2 is the mid-life stress amplitude and $K^{\prime }$ is the cyclic strength coefficient. It should be noted that the LCF life can be greatly affected by the presence of notches. Livieri et al. [70] considered a non-linear model to evaluate fatigue life of smooth and notched specimens. The authors showed a significant decrease in LCF life of samples with V- and U-notches compared with smooth specimens tested at *R* = −1. Thus, our further studies will aim to explore the influence of notches on the LCF life of this alloy.

**Figure 12:**
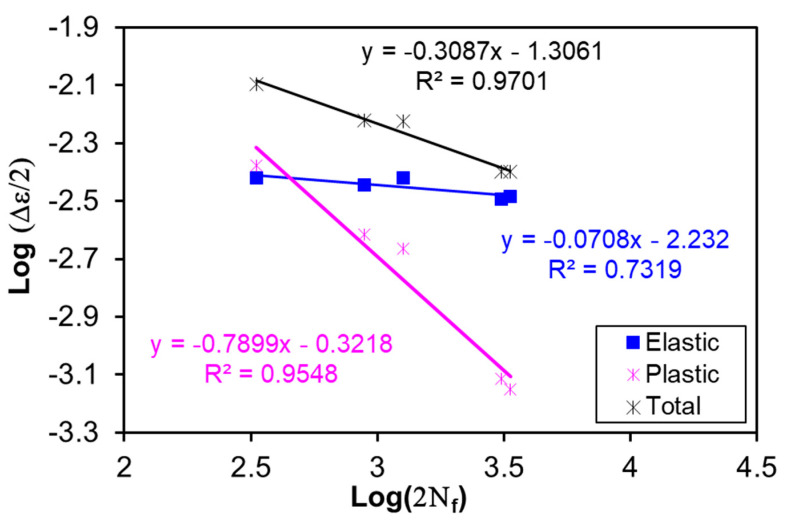
Strain-controlled fatigue life parameters of the heat-treated AlMgSiMnFe alloy.

**Figure 13:**
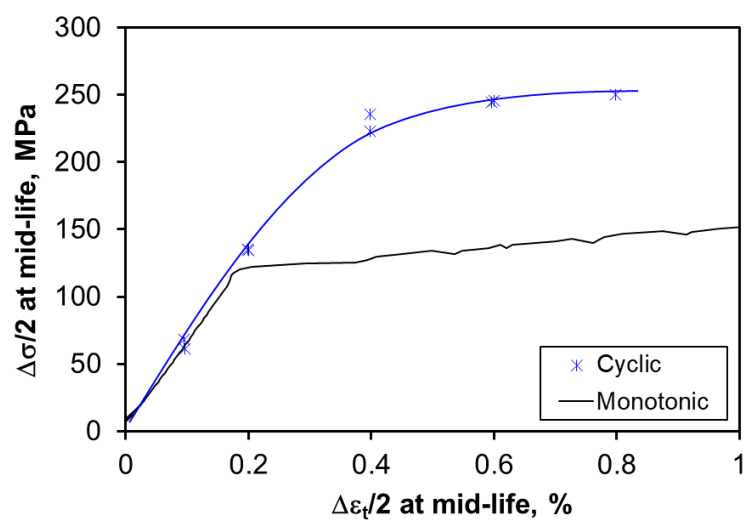
Cyclic stress–strain curve at mid-life of the heat-treated alloy vs. its monotonic tensile stress–strain curve.

The elastic, plastic and total strain amplitudes at the mid-life versus 2*N_f_* (the number of reversals to failure) of the heat-treated alloy are presented in Figure 12, and the evaluated fatigue parameters (*b*, *c*, *σ‘_f_*, *ε‘_f_*) are listed in Table 2. The monotonic and CSSC curves were plotted together in Figure 13, which show that the monotonic strength is lower than the cyclic strength of the heat-treated alloy. This reflects the occurrence of strong cyclic hardening, in agreement with Figure 9. The values of *K’* and *n’* following Equation (7) are also evaluated and listed in Table 2. Furthermore, the values of *b* and *c* calculated using Equations (5) and (6) proposed by Morrow [68] and Tomkins [69] were basically in agreement with the experimental values.

### 3.6. Fractography

**Figure 14:**
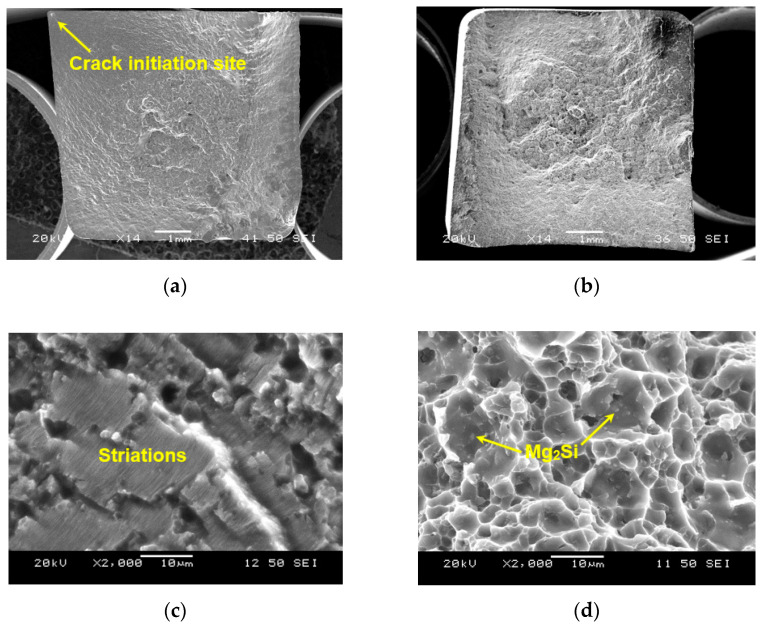
Fractographs of the samples fatigued at a strain amplitude of (**a**) 0.2% and (**b**) 0.6%, respectively; (**c**) fatigue crack growth region in the 0.2% sample and (**d**) final rapid fracture area in the 0.6% sample showing the embedded Mg_2_Si particles.

Casting defects such as porosity are normally present in many cast alloys that would be detrimental for their fatigue life because these locations act as stress concentration points, facilitating crack initiation and propagation, thus reducing the fatigue resistance of materials. For example, Yi et al. [71] demonstrated that the casting defects present in an Al-Si alloy greatly affect its fatigue properties. Figure 14a,b shows the overall fractographic images of the samples failed at a ∆ε_t_/2 of 0.2% and 0.6%, respectively. The crack initiated from the surface of the specimen, i.e., the near-surface porosity at the top left corner for∆ε_t_/2 = 0.2%, whereas the sample that failed at ∆ε_t_/2 = 0.6% appeared to have multiple sites of crack initiation from the specimen surface with a relatively smaller crack propagation area, due to a higher level of cyclic stress and strain experienced. Figure 14c shows characteristic fatigue striations in the crack propagation region on the fracture surface at a strain amplitude of 0.2%. It is known that the occurrence of fatigue striations was due to a repeated plastic blunting-sharpening process arising from the slip of dislocations in the plastic zone ahead of the fatigue crack tip [72]. To verify that Mg_2_Si particles are embedded in the α-Al grains, an image taken from the final fast fracture region at a strain amplitude of 0.6% is shown in Figure 14d. It is clear that numerous Mg_2_Si particles are uniformly embedded in the primary phase of the alloy, corresponding well to the OM and SEM observations of polished surfaces shown in Figure 2d and 3b. Therefore, the current heat-treated AlMgSiMnFe alloy is indeed a novel in-situ Mg_2_Si particulate-reinforced aluminum composite with a large amount of uniformly distributed spherical Mg_2_Si particles and a small amount of Fe- and Mn-containing particles. The observed Portevin–Le Chatelier effect (or serrated flow behavior) and cyclic hardening behavior of this alloy is mainly associated with the presence of these particles (Figure 2d, Figure 3b and Figure 14d).

## 4. Conclusions

Strain-controlled LCF tests were conducted on the heat-treated AlMgSiMnFe alloy under varying strain amplitudes in comparison with its as-cast condition.

- The microstructure of the alloy was composed of globular primary α-Al phase and alternating layered (α-Al + Mg_2_Si) eutectic structure plus a small amount of Al_8_(Fe,Mn)_2_Si phase in the as-cast condition, and abundant spherical Mg_2_Si particles evenly embedded in the α-Al matrix in the heat-treated condition. Like the as-cast alloy, no significant texture was observed in the heat-treated alloy.
- After heat treatment, the ductility and hardening capacity increased, although the YS and UTS decreased, exhibiting a normal strength–ductility trade-off relationship. This was primarily attributed to the spheroidization of intermetallic phases including both Mg_2_Si and Al_8_(Fe,Mn)_2_Si. The YS and UTS of the present heat-treated alloy were still much higher than those of a T6-treated similar (Al-5Mg-3Si) alloy reported in the literature.
- Serrated flow or Portevin–Le Chatelier effect was present in both tensile stress–strain curves and initial hysteresis loops of LCF tests as a result of DSA caused by the strong dislocation–precipitate interactions.
- The alloy exhibited strong cyclic hardening in both as-cast and heat-treated states beyond a total strain amplitude of 0.4%, below which cyclic stabilization sustained. The heat-treated alloy had a larger plastic strain amplitude and a lower stress amplitude at a given total strain amplitude and exhibited longer fatigue life in the LCF regime of higher total strain amplitudes.
- A new equation has been proposed to characterize the degree of cyclic hardening/softening on the basis of the stress amplitude of the first and mid-life cycles. The degree of cyclic hardening was observed to be higher in the heat-treated state than in the as-cast state.
- Fatigue crack initiation occurred from the surface of the specimen at lower strain amplitudes, while multiple crack initiation was observed at higher strain amplitudes. Crack propagation was characterized by typical fatigue striations, along with characteristic dimples and embedded Mg_2_Si particles especially observed in the final rapid fracture area.

## Figures and Tables

**Table 1:** Comparison of the tensile properties of the AlMgSiMnFe alloy in the heat-treated and as-cast conditions (strain rate: 1 × 10^−2^ s^−1^).

Condition	Yield Strength (MPa)	Ultimate Tensile Strength (MPa)	Elongation (%)	Strain-Hardening Exponent, n	Hardening Capacity	Hardness, HV
Heat-treated	122 ± 5.5	260 ± 0.9	15.7 ± 0.6	0.27	1.13	71.4 ± 2.7
As-cast [16]	185 ± 0.6	304 ± 0.3	6.3 ± 0.9	0.25	0.72	92.7 ± 1.6

**Table 2:** Low-cycle fatigue (LCF) parameters of the alloy in the heat-treated and as-cast conditions.

Low-Cycle Fatigue Parameters	Symbol	Heat-Treated Alloy	As-Cast Alloy [16]
Cyclic yield strength, MPa	*σ*‘_y_	228	240
Cyclic strain hardening exponent	*n*’	0.05	0.08
Cyclic strength coefficient, MPa	*K*’	340	445
Fatigue strength coefficient, MPa	*σ*‘_f_	324	425
Fatigue strength exponent	*b*	−0.04	−0.08
	*b* (Morrow)	−0.04	−0.06
	*b* (Tomkins)	−0.05	−0.07
Fatigue ductility coefficient	*ε*‘_f_	0.48	0.35
Fatigue ductility exponent	*C*	−0.79	−0.95
	*c* (Morrow)	−0.79	−0.72
	*c* (Tomkins)	−0.90	−0.87
